# Fungal infections in humans: the silent crisis

**DOI:** 10.15698/mic2020.06.718

**Published:** 2020-06-01

**Authors:** Katharina Kainz, Maria A. Bauer, Frank Madeo, Didac Carmona-Gutierrez

**Affiliations:** 1 Institute for Molecular Biosciences, NAWI Graz, University of Graz, Graz, Austria.; 2 BioHealth Graz, Graz, Austria.; 3 BioTechMed Graz, Graz, Austria.

**Keywords:** Candida, resistance, antifungals, yeast, antimycotics, drug, azoles

## Abstract

Annually, over 150 million severe cases of fungal infections occur worldwide, resulting in approximately 1.7 million deaths per year. Alarmingly, these numbers are continuously on the rise with a number of social and medical developments during the past decades that have abetted the spread of fungal infections. Additionally, the long-term therapeutic application and prophylactic use of antifungal drugs in high-risk patients have promoted the emergence of (multi)drug-resistant fungi, including the extremely virulent strain *Candida auris*. Hence, fungal infections are already a global threat that is becoming increasingly severe. In this article, we underline the importance of more and effective research to counteract fungal infections and their consequences.

Humankind has been plagued by infectious diseases throughout history, and the ongoing COVID-19 (Coronavirus disease 2019) pandemic is a daunting reminder that this susceptibility persists in our modern society. After all, communicable diseases remain one of the leading causes of death worldwide [1]. Unfortunately, some of these “microbial threats” have been underestimated and neglected by healthcare authorities, although they endanger millions of lives each year all over the world. Fungal infections (FIs) represent an example of such overlooked emerging diseases, accounting for approximately 1.7 million deaths annually [2]. To put these numbers in perspective, tuberculosis is reported to cause 1.5 million deaths/year [3] and malaria around 405,000 deaths/year [4]. The medical impact of FIs, however, goes far beyond these devastating death rates: FIs affect more than one billion people each year, of which more than 150 million cases account for severe and life-threatening FIs. Importantly, the number of cases continues to constantly rise [5]. Thus, FIs are increasingly becoming a global health problem that is associated with high morbidity and mortality rates as well as with devastating socioeconomic consequences [6].

A crucial factor that contributes to the rising number of FIs is the drastic increase of the at-risk population that is specifically vulnerable to FIs, including elderly people, critically ill or immunocompromised patients. The overall lifespan increase due to the achievements of modern medicine and social advancements, the growing numbers of cancer, AIDS and transplantation patients with the concomitant subscription of immune-modulating drugs as well as the excessive antibiotic use compose risk factors and niches for the development of FIs [7–9]. Furthermore, the increasing usage of medical devices such as catheters or cardiac valves leads to a higher risk for the formation of biofilms. Biofilms represent an assembly of highly diverse, complex and eminently organized cells embedded in an extracellular matrix that conveys protection from physical and/or chemical insults. Thus, biofilms are often resistant to existing treatments and, in fact, are considered to essentially contribute to the high mortality rates associated with invasive FIs [10, 11].

The acquired resistance to currently available antifungal drugs in previously sensitive strains as well as the increasing incidence of less susceptible fungal strains jointly constitute another decisive factor that contributes to the emergence of FIs. Although a number of pharmacological options for antifungal treatment do exist, they are currently limited to three distinct chemical classes: azoles, echinocandins and polyenes [5, 12]. Thereby, azoles represent the clinically most relevant subgroup, since most azoles show comparably high effectiveness, low toxicity, immunomodulatory capacity and the possibility of oral application [12, 13]. These advantages have encouraged their long-term therapeutic application and prophylactic use in high-risk patients, which in turn has propelled the acquisition of antifungal resistance against azoles and the rise of less susceptible strains. In addition, the usage of agricultural fungicides closely related to medically relevant antimycotics has boosted the environmental reservoirs for drug-resistant pathogens [5, 14]. Notably, the emergence of antimycotic resistance urged the US Center for Disease Control and Prevention (CDC) to rank drug-resistant *Candida* yeasts as “serious threats”, which represents the same threat level assigned to, for example, methicillin-resistant *Staphylococcus aureus* (MRSA) or vancomycin-resistant *Enterococci* (VRE) [15]. A menacing example for drug-resistant yeasts is *Candida auris*, ranked at an even higher threat level by the CDC (“urgent threat”) [15]. *C. auris* was first described in Japan in 2009 and has rapidly spread globally since then [8, 9]. *C. auris* seems to be significantly less susceptible to a number of standard healthcare disinfectants and can be easily transmitted from patient to patient, which is rather unusual for other fungal pathogens. Alarmingly, about 90% of all isolates are resistant to at least one class of antifungal drugs and about 30% to at least two classes. In 1% of the isolates, *C. auris* is resistant to all three classes of antimycotics [9, 15]. Unfortunately, multidrug resistance (MDR) is also gaining ground in other fungi, mostly *Candida* species (the most prevalent fungal pathogens). For example, in *Candida glabrata*, cross-resistance between azoles and echinocandins is being increasingly recognized [16]. The problem of MDR further restrains the already limited repertoire of treatment options, leaving some FIs *de facto* untreatable.

The efficiency of yeasts to develop resistance against fungicidal agents has been shown both by *in vitro* microevolution experiments as well as *in vivo* [17], and highlights the urgent need for novel approaches to combat fungal infections. In order to acutely overcome resistance development, new putative antifungal agents should preferably inhibit mechanisms that are known to confer resistance and/or target pathways that differ from those that are already engaged by commercially available medications (mostly involving the cell wall or the plasma membrane). An example of such efforts can be found in the current issue of *Microbial Cell*, in which Edouarzin and colleagues identified a compound (drimenol, a sesquiterpenoid primary alcohol that provides a promising starting point for the development of a novel antimycotic [18]. Among the tested drimane sesquiterpenoids, drimenol exhibited the most potent activity against different pathogenic fungi and was also active against fluconazole-resistant strains and *C. auris*. Moreover, the compound was also effective *in vivo*, as it conveyed protection against *C. albicans* in the *Caenorhabditis elegans* infection model. Intriguingly, drimenol might thereby act via a different mode of action than commonly used antifungals, as it seems to target protein trafficking, protein secretion and cell signaling [18]. However, further studies are needed to validate the feasibility of drimenol (and other drimane sesquiterpenoids) as putative therapeutic agents.

There is no doubt that the threat imposed by FIs will continue to increase worldwide with a number of obstacles (including resistance development) that need to be overcome. This demands rapid and innovative action at different levels. First, the search for therapeutic treatment options needs to be intensified: besides searching within the classical antifungal drug pipeline, novel therapeutic strategies might be found, for example, by modulating natural microbial competition within the microbiome or specific niches [19, 20]. Second, antifungal susceptibility testing needs to be further standardized, since the current lack of unified protocols causes discrepancies between laboratories and difficulties in the interpretation of obtained data [21]. Antifungal susceptibility testing is a crucial requirement to find the optimal treatment option for a patient as well as for the detection of antifungal resistance [5]. Third, the awareness for FIs at the social and governmental levels (and by extension healthcare authorities) should be raised. Even though FIs account for a tremendous number of (lethal) infections, their impact remains comparably underestimated. In sum, FIs are crucial contributors to the new old threat of infectious diseases, and upgrading our antifungal armamentarium by improving existing and/or devising novel antifungal strategies remains an urgent medical challenge.
